# Lithium and fluoxetine regulate the rate of phosphoinositide synthesis in neurons: a new view of their mechanisms of action in bipolar disorder

**DOI:** 10.1038/s41398-018-0235-2

**Published:** 2018-08-31

**Authors:** Adolfo Saiardi, Anne W. Mudge

**Affiliations:** Medical Research Council Laboratory for Molecular Cell Biology, University College London, Gower Street, London, WC1E 6BT UK

## Abstract

Lithium is widely used to treat bipolar disorder, but its primary mechanism of action is uncertain. One proposal has been that lithium’s ability to inhibit the enzyme inositol monophosphatase (IMPase) reduces the supply of recycled inositol used for membrane phosphoinositide (PIns) synthesis. This 28-year-old hypothesis is still widely debated, however, largely because total levels of PIns in brain or in cultured neurons do not decrease after lithium treatment. Here we use mature cultured cortical neurons to show that, although lithium has little effect on steady-state levels of either inositol or PIns, it markedly inhibits the rate of PIns synthesis. Moreover, we show that rapid synthesis of membrane PIns preferentially uses inositol newly imported from the extracellular space. Unexpectedly, we also find that the antidepressant drug fluoxetine (FLUO: Prozac) stimulates the rate of PIns synthesis. The convergence of both lithium and FLUO in regulating the rate of synthesis of PIns in opposite ways highlights PIns turnover in neurons as a potential new drug target, as well as for understanding mood control in BD. Our results also indicate new avenues for investigation of how neurons regulate their supply of inositol.

## Introduction

Bipolar disorder (BD) is a prevalent, costly and life-threatening illness, characterized by mood swings from depression to mania/hypomania, interspersed with periods of normal mood^1^. Lithium has been the major mood-stabilizing treatment for the disorder for 50 years. More recently, drugs developed for other illnesses have proved useful as mood stabilizers, including the anticonvulsants valproate (VPA) and lamotrigine (LTG), and various antipsychotics^2^. Antidepressant drugs are often used to treat depressive episodes, but they are usually given together with a mood stabilizer because they can destabilize the illness or precipitate a manic episode^3^. All the mood-stabilizing drugs have side effects that discourage bipolar individuals from taking them, so there is an urgent need to develop more specific drugs. In order to develop better drugs, there is first the need to understand the molecular basis of the illness. One-way forward is to analyse the relevant signalling pathways involved in lithium’s therapeutic action and to investigate why conventional antidepressant drugs destabilize mood in bipolar individuals but not in those with unipolar depression.

**Fig. 1:**
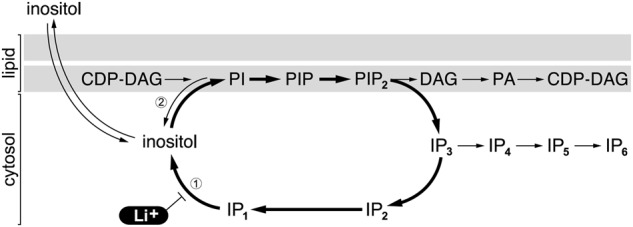
Schematic of the phosphoinositide (PIns) cycle. The key enzymes referred to in the article are indicated: (1) Inositol monophosphatase (IMPase), which is inhibited by lithium ions, and (2) Phosphatidylinositide synthase (PI-synthase) and the reverse phosphatidylinositol: inositol exchange reaction. The double arrows indicate the influx and efflux of inositol across the plasma membrane. Other abbreviations are: CDP-DAG cytidine diphosphate diacylglycerol, PI phosphatidylinositide, PIP phosphatidylinositide phosphate, PIP_2_ phosphatidylinositide-4,5-bisphosphate (this is the most abundant of the PIP_2_ species), DAG diacylglycerol, PA phosphatidic acid, IPs inositol phosphates with various numbers of attached phosphate groups, as indicated by the subscripts

There are two major candidate target proteins for lithium’s primary therapeutic action – inositol monophosphatase (IMPase), encoded by the gene *IMPA-1*^4^, which mRNA is enriched in neuronal axons^5^, and glycogen synthase kinase-3 (GSK-3)^6^. Both are inhibited at concentrations of lithium that approximate its therapeutic blood levels of 0.4–1 mEq/L, whereas the K_i_ for lithium’s inhibition of IMPase-2 is much higher than for IMPase-1 (10 mM vs 0.8 mM)^7,8^. Berridge, Downes and Hanley (1989)^9^ proposed that lithium’s ability to control BD depends on the inhibition of IMPase, which they suggested would decrease the supply of recycled inositol used for phosphoinositide (PIns) synthesis. This ‘inositol depletion’ hypothesis was attractive because it took account of the unusual uncompetitive kinetics of lithium’s inhibition of IMPase: inhibition is only seen when IMPase’s substrate, IP_1_, accumulates (see Fig. 1 for the PIns cycle and abbreviations). This suggested a mechanism in which lithium inhibition would be confined to neurons with high rates of PIns turnover, thus providing an explanation for the relative brain specificity of its action.

In support of this hypothesis, lithium has previously been shown to decrease steady-state levels of PIns in neurons: these effects, however, were only seen when PIP_2_ hydrolysis was hyper-stimulated for a prolonged period with the cholinergic agonist carbachol^10,11^, and this extreme pharmacological manipulation is unlikely to be relevant to normal brain function. Other studies that argue against a therapeutic role for IMPase inhibition have focussed on lithium’s effects on brain inositol and/or PIns levels. Lithium treatment transiently lowers brain levels of inositol (which are estimated to be ~6 mM) by ~25%, but it does not change brain PI or PIP_2_ levels^12,13^. Whole brain biochemical studies^14^ or inositol imaging of the brain^15,16^, however, gives no indication of how lithium might affect inositol and PIns metabolism in the sub-populations of neurons involved in mood control. Behavioural analysis of mice with targeted gene disruption of either inositol transporters or of IMPase are consistent with a lithium effect on the PIns cycle^17–19^, although the molecular basis of lithium’s action remains unclear. Given the controversy regarding lithium’s effects on ‘inositol depletion', the focus for lithium’s therapeutic action shifted to inhibition of GSK-3^8,20,21^.

Our previous work^22,23^ showed that lithium and anticonvulsant mood-stabilizing drugs have common inositol-reversible effects on neuron growth cone morphology. This finding re-kindled interest in inhibition of IMPase as a relevant therapeutic target for lithium, although a biochemical demonstration of an effect on PIns synthesis was missing. These findings led us to investigate both lithium’s and the antidepressant fluoxetine’s (FLUO: Prozac) action on PIns metabolism using neurons from rat cerebral frontal cortex, a brain region known to be involved in mood control.

These cultured cortical neurons do not express mRNA encoding the inositol synthesizing enzyme *myo*-inositol phosphate synthase (MIP-synthase)^22^, and so the neurons are dependent on the supply of inositol from the extracellular fluid. This arrangement is similar to that in vivo where MIP-synthase protein is confined to endothelial cells of the brain vasculature^24^. The cortical neurons also do not express mRNA encoding either of the two high capacity sodium-dependent *myo*-inositol transporters (SMITs-1 and 2), but they do express mRNA encoding the H^+^-dependent *myo*-inositol transporter (HMIT)^22,25^. In the brain, SMIT-1 mRNA is expressed mainly in astrocytes and the choroid plexus (but also by some neurons)^26,27^ (SMIT-2 mRNA is only in the meninges). The majority of neurons in the neocortex do express HMIT mRNA and protein, but Di Daniel et al.^28^ reported that cultured cortical neurons from mice with a null deletion of the HMIT gene import inositol from the extracellular space in a similar fashion to wild-type neurons. It is therefore unclear how these neurons take up inositol, which is a major hole in our current understanding of PIns metabolism in neurons.

It seems likely that the high concentrations of inositol in brain are located mainly in the non-neural cells expressing SMIT-1, SMIT-2 and MIP-synthase. Most previous studies of lithium’s effects in PIns synthesis used either cell lines that expressed the high capacity SMITs^29^ or cerebellum granule cell neurons in which the status of SMIT-1 was not documented^10,11^; these cells have mM levels of intracellular inositol^30,31^, which has contributed to the scepticism about inhibition of IMPase leading to inositol depletion as relevant to lithium’s therapeutic effects. Thus, a reinvestigation of the effect of lithium on inositol and PIns metabolism in primary neurons lacking SMITs is needed.

In this article, we show that large changes in extracellular and intracellular inositol levels have no effect on steady-state levels of PIns in cultured cortical neurons. Moreover, the majority of the inositol used for rapid synthesis of PIns is acquired from the extracellular space. We show that lithium has little or no effect on steady-state levels of either cytosolic inositol or membrane PIns. In contrast, lithium markedly inhibits the rate of PIns synthesis: we propose that this inhibition is due, in part at least, to the accumulation of IP_1_. We also show that FLUO markedly decreases cytosolic inositol levels without affecting steady-state PIns levels, but that FLUO increases the use of recycled inositol for PIns synthesis.

## Materials and methods

### Cell culture

The cerebral cortices were dissected from E17 Sprague-Dawley rats, collected in Ca^++^-free–phosphate-buffered saline, and dissociated mechanically to single cells. Cells were plated in 6- or 12-well plates (Nunc) (pre-coated with poly-d-lysine) at ~1–2 × 10^6^ cells per well and grown in Neurobasal medium supplemented with B27, GlutaMAX-1, penicillin/streptomycin and nerve growth factor (7S form NGF: 50 ng/ml) (all from Invitrogen) at 37 °C and with 6% CO_2_. Cytosine arabinoside (ara-C) was added at 5 × 10^−6^ M between days 1 and 5 to eliminate non-neural cells; the addition of NGF allowed us to completely replace the growth media after ara-C treatment and to replace 50% 1 day prior to the experiment on day 11 (D11). Cultures were checked by microscopy before use to ensure that there were no visible flat cells; the flat cells seen in D11 cultures without ara-C were identified as astrocytes by immunohistochemistry.

### Inositol labelling

PIns and inositol in neurons were labelled to steady state between days 6 and 11 (D6–11) with 5–10 μCi/ml of ^3^H-*myo*-inositol added to the media (specific activity 30 Ci/mmol: American Radiochemicals Inc.). (Note: we refer to *myo*-inositol as simply inositol elsewhere in this article.) For rapid synthesis and influx experiments, ^3^H-inositol was added for 1 h. For efflux experiments, neurons were labelled for 5 days (D6–11), then washed rapidly; these neurons were re-fed with growth medium, with or without drugs, and returned to the CO_2_ incubator at 37 °C (for 1 h or as stated) before collecting the media for counting (efflux) and then extracting the cytosol and membranes sequentially.

### Washing

Radioactivity was removed from the extracellular space by washing (3 × 2–3 ml) with a salt solution based on the salt composition of Neurobasal medium^32^, except that the NaHCO_3_/CO_2_ buffer was replaced by 10 mM HEPES: 5 mM NaOH (pH 7.4 in air) and the osmolarity due to the B27 additive was compensated for with additional NaCl; total osmolarity of the wash solution was 270 mOs cf. Neurobasal medium 274 mOs. The wash solution was warmed to 37 °C before use.

### Extractions

Acid soluble and membrane PIns were extracted and analysed by strong anion-exchange high-pressure liquid chromatography (SAX-HPLC) using a modification of the methods described by Azevedo and Saiardi^33^ and Dove and Mitchell^34^ and in Supplemental Methods. Briefly, 2 M perchloric acid was used to extract components in the cytosol: these extracts would also contain acid-soluble components from intracellular compartments and components attached to membranes, but for convenience we refer to all the acid-soluble components as cytosol in the text and in the figures. The cell membranes were then scraped off with an acidic/methanol solution and the lipids extracted with chloroform: two phases were generated, the chloroform layer containing the lipid PIns and the polar layer, which had minimal radioactivity; there was also a white interphase layer of unknown composition. In 5-day labelling experiments, the interphase layer contained ~10% of the radioactivity found in the lipid extracts and this was not affected by drug treatments. In 1-h labelling experiments, the radioactivity in the interphase layer was negligible.

For the flux experiments, cytosolic components were extracted in 2 M perchloric acid (2 × 200 μl/well for a 12-well plate) and the lipid membranes were then scraped off in 0.1 M NaOH/0.1% Triton-X100 (2 × 200 μl/well) and the lipid extracts allowed to dissolve in capped scintillation tubes over 2 days at 20 °C before the addition of 5 ml of scintillation fluid (Ultima-Flo AP LCS-mixture; Packard). The counting efficiency of a constant amount of radioactivity was within ± 10% in the acid or basic extracts and in the Neurobasal medium. In 5-day labelling flux experiments, the radioactivity in the NaOH extracts were not corrected for the presence of the non-lipid components (~10% total cpm). Radioactivity was expressed routinely as counts per minute per well (cpm/well).

### Statistical analyses

Data were analysed where appropriate by one-way analysis of variance followed by unpaired *t*-test. Data from individual experiments are presented in the figures as either means ± the standard deviation (SD) or ± the standard error of the mean (SEM) as indicated, where the '*n*' refers to the number of wells used for each condition; each composite shows data from neurons plated at the same time except for Fig. 4 where a and b were from a separate plating to those used for c and d. The average changes calculated from similar experiments using neurons from separate platings are presented in the text as mean ± SEM and the number of separate experiments indicated. Statistically significant differences from controls are indicated on the figures by *(*p* < 0.05), **(*p* < 0.01) or ***(*p* < 0.005).

## Results

Rat cortical neurons were plated and grown at a constant number per well and cultured in defined medium for 10–12 days. At this point, the axon outgrowth was stable, there were many synaptic profiles seen by electron microscopy (Supplemental Figure S1), and there were no visible non-neural cells. These cultures have been shown to contain glutaminergic and GABAergic neurons and to be electrically active after ~ day 8. Inositol uptake was not inhibited by phloridzin in the dose range 30–600 μM, which is known to inhibit both SMITs-1 and -2^31,35^. We added ^3^H-inositol to the growth medium, which was taken up by the neurons and incorporated into their membranes. Because the neurons do not synthesize inositol, the levels of radioactivity in cytosolic ^3^H-inositol, ^3^H-IPs and membrane ^3^H-PIns (expressed as cpm/well) reflect the relative total amounts of each of these components.

### Extracellular inositol does not influence the levels of PIns

**Fig. 2:**
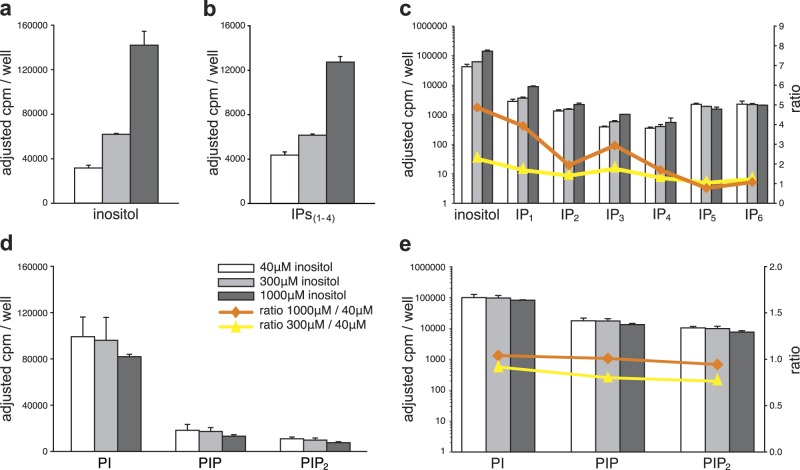
Relative steady-state levels of inositol, IPs and lipid PIns in cortical neurons cultured in various levels of inositol. Neurons were grown for 5 days (D6–11) in various concentrations of inositol as shown, together with ^3^H-inositol (10 µCi/ml). Histograms show the cpm/well in fractions from SAX-HPLC analysis (see also Figure S2). Data were normalized for constant specific activity of ^3^H-inositol by dividing the cpm by 7.5 (for 40 μM inositol) and multiplying the cpm by 3.3 (for 1000 μM inositol). Therefore, the *Y* axis (adjusted cpm/well) indicate the cpm corrected for specific activity. Data shown are the mean and range from duplicate samples; the experiment was repeated with similar results. **a** Relative levels of cytosolic inositol and **b** relative levels of IPs_(1–4)_. **c** Same data as in (**a**, **b**) but shown on a log_10_ scale. The ratios of each component at the different concentrations of inositol are indicated by the lines. **d**, **e** Relative levels of membrane PIns extracted from the same neurons used to generate data in (**a**), (**b**) and (**c**) shown on a linear (**d**) and a log_10_ scale (**e**). The ratios of each component at the different concentrations of inositol are indicated by the lines as shown

We first investigated how extracellular inositol levels affect the steady-state levels of cytosolic and membrane inositides by culturing neurons for 5 days in medium containing ^3^H-inositol and one of three concentrations of inositol: 40 μM (inositol concentration in Neurobasal medium and approximate blood level) or 300 μM or 1000 μM [estimated cerebral spinal fluid (CSF) levels are ~130 μM^36,37^]. After washing, we extracted the neurons sequentially with acid (for cytosolic components) followed by methanol/chloroform (for membranes), and analysed the relative amounts of ^3^H-labelled components in each extract using SAX-HPLC (see Supplemental Figure S2). The 7.5- and 25-fold changes in extracellular inositol caused only 1.9- and 4.5-fold changes, respectively, in intracellular levels of ^3^H-inositol, with smaller changes in the levels of individual cytosolic IPs_(1–4)_ (no changes occurred in the levels of IPs_(5, 6)_) (Figs. 2a, b). In contrast, there were no changes in the levels of membrane PIns (^3^H-PI, ^3^H-PIP or ^3^H- PIP_2_) in response to the 4.5-fold change in intracellular inositol and the 25-fold change in extracellular inositol (Figs. 2d, e). These results indicate that neither the extracellular nor the intracellular concentration of inositol is the major regulator of steady-state levels of PIns in cortical neurons.

Note that, in this experiment and others after steady-state labelling for 5 days, the ratio of ^3^H-inositol incorporated into membranes to the amount in the cytosol (using media with 40 μM inositol) was on average 4.9 ± 0.7 (*n* = 5 experiments). When hydrolysis of PIP_2_ was stimulated with either carbachol or by membrane depolarization with 40 mM KCl to produce IP_3_, the majority of this IP_3_ was rapidly converted to IP_2_, IP_1_ and inositol within 30 s (Supplemental Figure S3a): this recycling of inositol from PIns is referred to as ‘the PIns cycle’ and is indicated by the bold arrows in the diagram (Fig. 1). After 20 min of stimulation, all of the IP_3_ was converted to IP_2_, IP_1_ and inositol with a small fraction converted to IP_4_ (Supplemental Figure S3b). In 5-day steady-state ^3^H-inositol labelling experiments, electrical activity in the neurons drives PIns hydrolysis with the majority of IP_3_ dephosphorylated but a small fraction of IP_3_ phosphorylated to generate labelled IPs_(4–6)_.

### Lithium and FLUO effects on steady-state levels of inositol and PIns

**Fig. 3:**
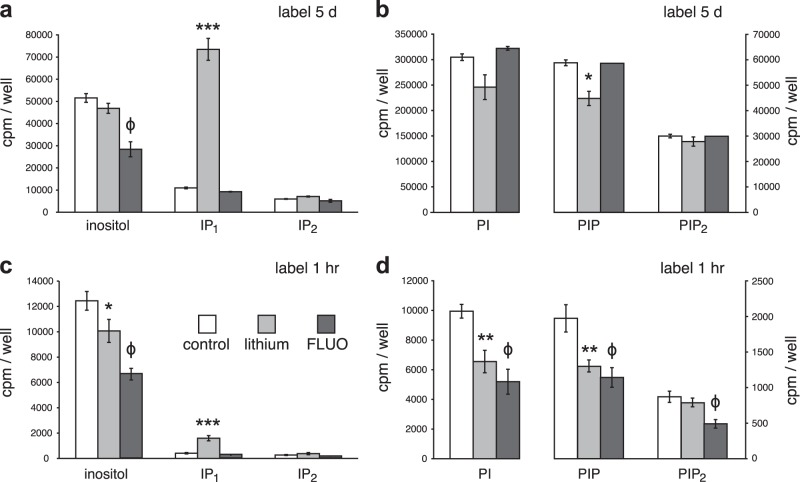
Effect of lithium and FLUO on steady-state levels of PIns and the rate of PIns synthesis. **a** Neurons were labelled to steady-state for 5 days (D6–11) with ^3^H-inositol (10 µCi/ml) in presence of 300 μM inositol and treated for 20 h with 1 mM lithium or 3 μM FLUO as indicated prior to acid extraction of the cytosol. Histograms show the cpm/well in fractions from SAX-HPLC analysis. Data are the mean and range from duplicate samples. The symbol *** indicate a highly significant (*p* < 0.005) increase in IP_1_ after lithium treatment compared with controls and the symbol (ϕ) indicates a highly significant (*p* < 0.005) decrease in inositol levels after FLUO compared with controls. Data shown are the mean ± SD, *n* = 3. **b** Lipids were extracted from the same neurons used for (**a**) and processed for chromatography of the glycerophosphoinositide head groups. Histograms show the cpm/well in fractions from SAX-HPLC analysis. Data are the mean and range from duplicate samples. The symbol * indicates a significant (*p* < 0.05) decrease in PIP due to lithium; similar data were obtained in three separate experiments. **c** Neurons from the same plating as those used to generate data in (**a**, **b**) were labelled for 1 h with ^3^H-inositol prior to acid extraction of the cytosol. Neurons were also treated for 20 h prior to extraction with 1 mM lithium or 3 μM FLUO as indicated. Histograms show the cpm/well in fractions from SAX-HPLC analysis. Data shown are the mean ± SD, *n* = 3. The symbols * and *** indicate a significant (*p* < 0.05) or highly significant difference (*p* < 0.005), respectively, with lithium treatment compared with controls. The symbol (ϕ) indicates a highly significant (*p* < 0.005) decrease in inositol levels of 45% with FLUO treatment compared with controls. **d** Lipids were extracted from the same neurons used for (**c**) and processed for chromatography of the glycerophosphoinositide head groups. Histograms show the cpm/well in fractions from SAX-HPLC analysis. Data shown are the mean ± SD, *n* = 3. The symbols ** indicate very significant differences (*p* < 0.01) with lithium treatment compared with controls. Similar data were obtained in three separate experiments. The symbols (ϕ) indicates a highly significant (*p* < 0.005) decrease in levels of PI, PIP and PIP_2_ with FLUO treatment compared with controls; these decreases were all equivalent to the ~45% decrease in inositol shown in (**c**)

We analysed the steady-state levels of inositol, IPs and PIns after treating the neurons with either 1 mM lithium or 3 μM FLUO for 20 h. As shown in Figs. 3a, b, lithium had no significant effect on the level of inositol, but it induced a small decrease in PIP. Lithium, however, caused a marked accumulation of IP_1_, the substrate for IMPase, such that its level was 1.6-fold greater than that of inositol (Fig. 2a). Lithium also inhibits inositol polyphosphate-1-phosphatase (IPPase) with uncompetitive kinetics (K_i_ 0.3 mM)^8,38^, but the increase in the level of its substrate, IP_2_, was small (Fig. 3a); there was no accumulation of IP_3_ or of IP_(4–6)_ as suggested by Sade et al^39^. In three separate experiments, the increase in the level of IP_1_ after lithium treatment was 8 ± 0.8-fold compared with a 1.7 ± 0.07-fold increase in IP_2_.

**Fig. 4:**
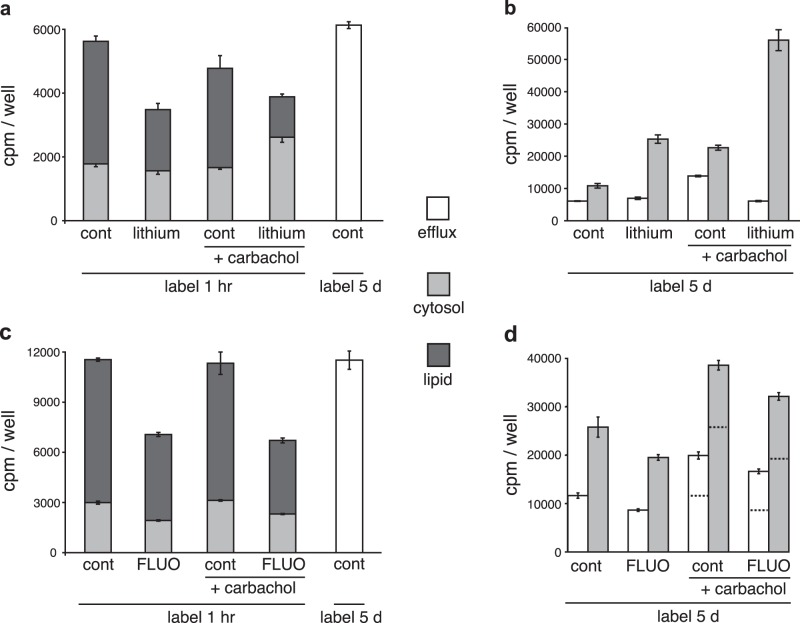
The flux of inositol across the plasma membrane and the effect of drugs. **a**, **c** Neurons were treated with either 1 mM lithium or 3 μM FLUO for 20 h before extraction, and also labelled with ^3^H-inositol for the last 1 h. Some neurons were also stimulated with carbachol (1 mM) during the 1-h labelling period. The histograms show the cpm/well in extracts of the cytosol (light grey bars) and extracts of membranes from the same neurons (dark grey bars). Data are means ± SEM, *n* = 4. Data in (**a**–**b**) and (**c**–**d**) are from two separate platings and each experiment was repeated with similar results. For comparison of influx and efflux, the white bar shows the efflux from control neurons as shown in (**b**) and (**d**) but plotted on the same scale as the cytosol and lipid. **b**, **d** Neurons were labelled to steady-state for 5 days (D6–11) with ^3^H-inositol and treated with either 1 mM lithium or 3 μM FLUO for the last 20 h before washing away the radioactivity, re-feeding the neurons with media and drugs, and returning the neurons to the CO_2_ incubator for 1 h. Some neurons were also stimulated with carbachol (1 mM) during the 1-h incubation. The white bars show the amount of inositol present in the collected media after 1 h. The light grey bars show the amount remaining in the cytosol (acid extraction) of the same neurons. Data are means ± SEM, *n* = 4

FLUO consistently decreased the levels of intracellular inositol to a much greater extent than lithium (by 45% in Fig. 3a), but it had no effect on the steady-state levels of PI, PIP or PIP_2_ (Fig. 3b); other examples of FLUO effects are shown in Figs. 4c, d and Supplemental Figure S5. These results with FLUO argue strongly against the notion that lithium inhibits PIns synthesis because of ‘inositol depletion’ in these neurons, as originally suggested by Berridge et al.^9^; they are also consistent with our finding that intracellular inositol levels do not determine the steady-state levels of PIns in these neurons.

### PIns metabolism in neurons

We next looked at the effects of lithium and FLUO on the rate of synthesis and degradation of PIns as it was effects on turnover of PIns rather than their steady-state levels that first attracted the attention of Hokin and Hokin and led to the discovery of the PIns signalling system^40^. We exposed the neurons to ^3^H-inositol for short periods (10 min, 1 h and 3 h) before extracting and analysing the incorporation of ^3^H-inositol into lipid PIns and degradation products in the cytosol. We found that both uptake of inositol and its incorporation into membranes increased from 10 min over 3 h: ^3^H-IP_1,2_ were detected in the cytosol after 10 min, ^3^H-IP_3,4_, after 1 h, whereas ^3^H-IP_5_ was detected after 3 h (Supplemental Figure S4). We decided to use a 1-h exposure to assess the rate of inositol uptake and synthesis of PIns after drug treatment.

### Lithium inhibits the rate of PIns synthesis

We tested the two drugs on the rate of PIns synthesis using neurons from the same plating as those used for steady-state labelling in Figs. 3a, b. As shown in Figs. 3c, d, ~50% of the ^3^H-inositol taken up into the neurons in 1 h was incorporated into membranes, and radioactivity was also found in cytosolic IP_1_ and IP_2_ (Figs. 3c, d). In contrast to measuring steady-state levels (5 days labelling), lithium very significantly (*p* < 0.01) decreased the incorporation of ^3^H-inositol into both PI and PIP by 34% (assessed relative to control), although incorporation into PIP_2_ was not affected (Fig. 3d): there was a 19% decrease in the level of ^3^H-inositol and an equivalent increase in the level of ^3^H-IP_1_ such that total cytosolic radioactivity was unaffected by lithium (Fig. 3c). Similar results were obtained in four separate experiments, and the average inhibition of the rate of incorporation of inositol into membrane PIns by lithium was 46 ± 4 % (see also Fig. 4a).

FLUO decreased the level of ^3^H-inositol in the cytosol after 1 h by 46% (Fig. 3c), which is equivalent to the FLUO-induced decrease in steady-state levels of inositol in neurons from the same plating (Fig. 3a). In contrast to the lack of effect on steady-state levels of PIns, FLUO inhibited the rate of incorporation of ^3^H-inositol into PI, PIP and PIP_2_ compared with controls; each of these highly significant (*p* < 0.005) decreases indicated by the symbol (ϕ) was directly proportional to the decrease in the steady-state level of inositol (the average decrease in the five components in Figs. 3a, c, d was 45 ± 2%). These results with FLUO are in contrast to the effect of lithium, where the decreases in the rate of incorporation of ^3^H-inositol into PI and PIP (each 34%) occurred without a change in total cytosolic inositol plus IP_1_ and with no change in the steady-state level of cytosolic inositol.

### Inositol rapidly crosses the plasma membrane

We then looked in more detail at the flux (in and out) of ^3^H-inositol across the plasma membrane. For influx, we labelled for 1 h and then washed the neurons and extracted the cytosol and membranes sequentially. For efflux, we labelled to steady state for 5 days with the same specific activity of ^3^H-inositol, using neurons from the same plating as those used for influx; we washed the neurons, and after 1 h collected the medium (efflux), before extracting the cytosol and membranes sequentially. We quantitated the radioactivity without separating components by chromatography. As shown for lithium in (Figs. 4a, b) and FLUO in (Figs. 4c, d), the majority of ^3^H-inositol influx in control neurons was incorporated into membranes in 1 h (66 and 74 % respectively, first bars in Figs. 4a, c). Strikingly, the efflux of ^3^H-inositol in 1 h (white bars) from control neurons was perfectly matched to the total influx (cytosol plus lipid) of untreated controls. As shown in the controls in Figs. 4b, d (steady-state labelling for 5 days), the efflux was ~50 % of the total soluble pool of ^3^H-inositol and ^3^H-IPs (light grey bars). In other similar experiments, the average proportion of influx incorporated into lipid in 1 h was 69 ± 1.5% (*n* = 5) and the average efflux compared with the cytosolic steady-state labelling radioactivity was 50 ± 2.6% (*n* = 4). The rapid exchange of cytosolic inositol with extracellular inositol, together with the high proportion of newly acquired inositol that is incorporated into membrane, indicates that rapid PIns synthesis depends primarily on inositol newly imported from the extracellular space. These data also suggest that the pool of cytosolic inositol in regions of rapid PIns turnover may be negligible.

In order to increase turnover of PIns, we stimulated the neurons with the muscarinic agonist carbachol. This increased both the cytosolic level of ^3^H-inositol and ^3^H-IPs, as well as the efflux by ~2-fold (Figs. 4b, d), the proportion of inositol generated by carbachol-stimulated hydrolysis of PIP_2_ that appeared in the efflux was 47 ± 4%. This indicates that efflux is concentration dependent and, surprisingly, that much of the inositol coming from PIP_2_ hydrolysis equilibrates rapidly with extracellular inositol. We confirmed that PIP_2_ hydrolysis was also stimulated by membrane depolarization with 40 mM KCl as previously reported^41^ (see Supplemental Figure S3), and noticed that the rate of inositol efflux increased with this stimulation as it did with carbachol (data not shown).

### Drugs have indirect effects on inositol uptake by neurons

We found no evidence to indicate that the inhibitory effects of either lithium or FLUO on inositol uptake were due to direct inhibition of transport. Lithium did not change either the total cytosolic content of ^3^H-inositol (Fig. 3a) or the efflux (Fig. 4b), although it greatly decreased the incorporation of ^3^H-inositol into lipid PIns (Fig. 4a); this indicates that lithium’s inhibition of inositol influx is indirect and is linked to the rate of PIns synthesis. Carbachol stimulation decreased lipid PIns and increased both cytosolic content and efflux of ^3^H-components (Fig. 4b), indicating that much of the inositol recycled from PIns hydrolysis equilibrates with the extracellular space. The large increase in IP_1_ resulting from lithium inhibition of PIns synthesis during carbachol stimulation did not change inositol efflux relative to unstimulated control efflux (Fig. 4b).

Treatment with FLUO, however, decreased both influx (Fig. 4c) and efflux of ^3^H-inositol in the same proportion to the decrease in cytosolic ^3^H-inositol (Fig. 4d). Intriguingly, the increased efflux due to carbachol stimulation of PIP_2_ hydrolysis was almost identical ( ±3 %) in FLUO-treated neurons compared with controls (Fig. 4d). These last results indicate that FLUO, as with lithium, does not inhibit efflux directly.

The results with lithium and FLUO treatment confirm the effects of these drugs on inhibiting the rate of PIns synthesis and inositol uptake, respectively, indicating that this simplified influx experiment may be useful in drug screening compared with the CDP-DAG experiments used in the past^10,28^.

Indeed, we used the influx of ^3^H-inositol to assess the dose responses to drugs when added for 20 h prior to the transport studies. We found significant effects at <1 mM for lithium and <1 μM for FLUO (Supplemental Figure S5). Lithium also inhibits the enzyme GSK-3, so we tested a small-molecule inhibitor of GSK-3 – SB415286 (a gift from GlaxoSmithKline, Harlow, UK) – in this uptake assay using a dose that inhibits 45% of neuronal GSK-3 activity^42^. SB415286 had no effect on either inositol uptake or PIns synthesis (data not shown), indicating that lithium inhibition of GSK-3 does not contribute to the decrease in the rate of PIns synthesis.

### FLUO stimulates incorporation of recycled inositol into PIns

**Fig. 5:**
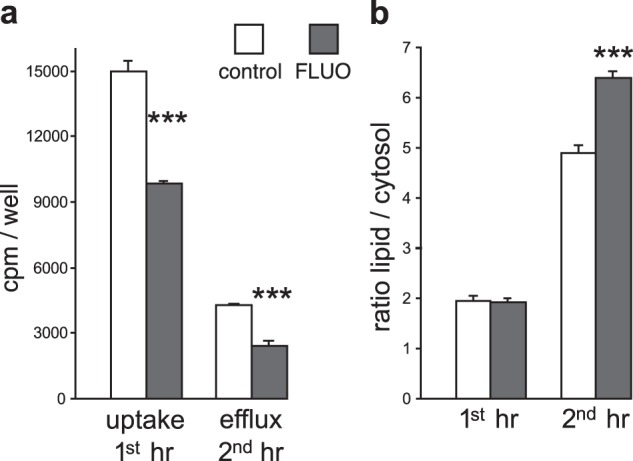
FLUO effects on the incorporation of recycled inositol into lipid PIns. **a** Histograms on the left show the total uptake (cytosol plus membrane) of ^3^H-inositol in 1 h in control compared with 3 µM FLUO-treated neurons as indicated. Histograms on the right show the efflux from neurons in the second hour after washing away the radioactivity. The symbols indicate highly significant (*p* < 0.005) decreases in FLUO-treated neurons compared with controls. Data are means ± SEM, *n* = 4. **b** The ratio of radioactivity in the membranes to that in the cytosol after 1 h compared with the ratio after 2 h. The same neurons generated the left and right histogram pairs in both (**a**) and (**b**). The increase in the ratio in the second hour for FLUO-treated neurons compared with controls was highly significant (*p* < 0.005) as indicated ***

As ^3^H-inositol influx and efflux were both decreased in the presence of FLUO, whereas steady-state levels of PIns were the same as controls, we investigated whether FLUO stimulates incorporation of recycled inositol into PIns, thereby decreasing the need for additional uptake of inositol. We labelled neurons for 1 h and found that the total influx of ^3^H-inositol was decreased by 34% in FLUO-treated neurons compared with controls (Fig. 5a); the ratio of membrane to cytosolic radioactivity in FLUO-treated neurons compared with controls was identical (1.93 vs 1.96: Fig. 5b). We washed away the extracellular ^3^H-inositol from a second set of neurons from the same plating and incubated them for a further hour. After the second hour, the cytosolic content of ^3^H-inositol in control neurons was decreased as expected, but cytosolic content and efflux of ^3^H-inositol in FLUO-treated neurons was further decreased to ~43% that of controls (Fig. 5a). As predicted, there was a highly significant (*p* < 0.005) 30% increase in the ratio of membrane to cytosolic radioactivity in FLUO-treated neurons compared with controls in the second hour (Fig. 5b). This result is consistent with the notion that FLUO stimulates greater incorporation of recycled inositol into PIns compared with controls. The apparent inhibitory effect of FLUO on synthesis of PIns as assessed by incorporation of ^3^H-inositol into PIns is therefore due to the decrease in inositol influx and efflux. Instead, the shift to the use of recycled inositol indicates a stimulatory effect of FLUO on the rate of PIns synthesis.

## Discussion

Much of the 28-year debate about lithium’s inhibition of IMPase as a possible therapeutic target has focussed on lithium’s effects on regulation of inositol and PIns steady-state levels in the brain or in neurons. As noted previously, it was the rate of synthesis and not the levels of PIns in pancreatic slices that initially attracted the attention of Hokin and Hokin^40^ and led to the discovery of the PIns cycle and its importance in intracellular signalling^43,44^. Our finding that lithium causes a marked inhibition of the rate of synthesis of PIns, and hence turnover of PIP_2_, without affecting steady-state levels refutes one of the main arguments against IMPase being a relevant primary therapeutic target of lithium in the treatment of BD. The difference between lithium effects on steady-state levels and the synthesis rate in neurons suggests that there may be more than one pool of PIP_2_ with different rates of synthesis, which would not be surprising given the elaborate structure of neurons illustrated in our electron micrographs and the ubiquitous PIP_2_ and InsP signalling network. In addition, if the rates of PIP_2_ hydrolysis and synthesis are matched, as indicated by Batty and Downes^29^, then the turnover rate in any compartment could change without producing a change in steady-state levels.

A large accumulation of IP_1_ is taken as an indication of rapid turnover of PIns^45^, which we attribute to the electrical activity in our cultured neurons, and which may account for the difference between our results and those of del Rio et al.^10,41^. It is striking that the average 8-fold increase in levels of accumulated IP_1_ resulting from lithium treatment is 1.6-fold higher than the levels of intracellular inositol. We therefore suggest that the therapeutically relevant consequence of lithium’s inhibition of IMPase is not to limit the availability of recycled inositol for PIns re-synthesis, as originally suggested^46^: rather, it is the accumulation of IP_1_, which then inhibits PIns synthesis. The ability of inositol to reverse the effects of lithium could then be explained by the need for additional inositol to overcome the inhibition of PI-synthase by IP_1_. We have preliminary evidence that IP_1_ does inhibit PI-synthase in membrane extracts. This enzyme, however, is unusual in that it also catalyses the reverse phosphoinositol:inositol exchange reaction^47,48^ and so a full analysis of the inhibition is outside the scope of the present article.

Although the levels of inositol in human ventricular CSF are estimated to be in the range of 110–160 μM^36,37^, it is not known what the levels are in the restricted extracellular spaces surrounding different neurons and in the different compartments of individual neurons. It is likely that these inositol levels may become limiting, particularly in regions of neurons that have rapid PIns turnover – for example, synapses or growth cones. Thus, lithium’s effect mediated by IP_1_ inhibition of PI-synthase could be more pronounced in specific neuronal compartments, as well as in specific neurons, even if lithium inhibits IMPase and causes IP_1_ accumulation in all neurons. Indeed, inositol-reversible effects of lithium and anticonvulsant mood stabilizers on neuronal function have only been observed in specialized neuronal structures such as growth cones^23^ and synapses^49^.

Our study also shows that ~50% of the total intracellular pool of inositol, including that generated by PIP_2_ hydrolysis, is exchanged with extracellular inositol each hour and that rapid PIns synthesis preferentially uses newly imported inositol. These results add further support to the idea that the therapeutically relevant consequence of lithium’s inhibition of IMPase is not to limit the availability of recycled inositol, which has been the subject of much research. In addition, our study looked specifically at rapid turnover of ^3^H-PIns as we measured labelling in neurons after 1 h, whereas studies such as with brains of *IMPA-1* ^−/−^ mice looked at labelling of PIns after 24 h^39^, which would include many cell types, as well as many pools of neuronal PIns.

It is important to note that vesicle release is triggered by extracellular Ca^++^ influx via channels^50^ and that PIP_2_ signalling, rather than IP_3_ signalling, plays a crucial role in regulating vesicle recycling processes involved in synaptic plasticity^51,52^, as well as activity of many ion channels^53^. Lithium seems to indirectly decrease inositol influx into the neurons, however, which may be due to a close link between uptake of inositol and PIns turnover. The rapid flux of inositol we describe in our electrically active neurons raises the possibility that inositol may be acquired from the extracellular space via synaptic vesicle recycling rather than by a transporter. PI-synthase is located in the endoplasmic reticulum^54^ and PIns is then inserted into the plasma membrane by a fusion process: linking the supply of inositol to PIns turnover in synapses via vesicle recycling is therefore an intriguing notion. Note also that HMIT is located in an endosomal compartment^25,28^, where it may supply inositol for PI-synthase.

In the context of new drug development, there has been recent emphasis on lithium’s ability to inhibit GSK-3 rather than IMPase as the most relevant therapeutic primary drug target^8,21,55^, although no GSK-3 inhibitors have progressed to clinical trials for BD. We previously showed that the inositol-reversible effects of lithium on neurons are not mimicked by GSK-3 inhibitors, although such inhibitors do mimic other neuronal effects of 10 mM lithium^22^. In the present study, we show that GSK-3 inhibition does not mimic lithium’s inhibition in PIns synthesis, confirming that these two targets of lithium are not necessarily linked. Our present results with both lithium and FLUO, together with our discovery that neurons use extracellular inositol for rapid PIns turnover, should re-establish PIns turnover as a primary drug target for BD treatment. Atack et al.^56^ designed drugs based on the structure of IP_1_ to inhibit IMPase, although these drugs failed to reach clinical trials due to delivery problems. Our suggestion that IP_1_ inhibits PI-synthase makes this second enzyme a possibility for new drug screening studies. A recent drug screen identified a new IMPase inhibitor Ebselen^57^, but its ability to mimic lithium effects on IP_1_ accumulation or PIns turnover has yet to be established, surprisingly. The large accumulation of IP_1_ in response to lithium results from the mechanism whereby lithium only binds to IMPase when the substrate is bound, resulting in the unusual uncompetitive inhibition. It seems unlikely that a non-competitive inhibitor such as Ebselen would mimic this crucial aspect of lithium’s inhibition. Lithium has recently been shown to change the excitability of neurons derived by stem cell technology from lithium-responsive and non-responsive individuals^58^: It would be interesting to know whether this effect can be reversed by inositol vs. GSK-3 inhibitors as PIP_2_ is known to regulate voltage-sensitive channel properties^53^.

To our knowledge, the finding that FLUO has a major effect on inositol levels and PIns synthesis at doses of < 1 µM has not been described previously. We initially tested FLUO in our growth cone assay because of our interest in FLUO’s destabilizing effects on mood in BD, and the results suggested that FLUO might have effects on the PIns cycle. In the present experiments, we first used FLUO as a tool to manipulate inositol levels in order to explore inositol’s role in regulating PIns synthesis in neurons, just as lithium was used to manipulate the levels of IPs in early experiments on IP_3_ signalling^59^. Our finding, however, that FLUO stimulates PIns turnover by increasing the incorporation of inositol generated by PIP_2_ hydrolysis into PIns re-synthesis offers an explanation for FLUO’s ability to cancel out the effects of lithium and VPA on growth cones, where we suggest that inositol uptake capacity may be limited. We found significant drug effects at < 1 mM for lithium and < 3 µM for FLUO (Supplemental Figure S5). Both concentrations are therapeutically relevant: lithium blood levels are 0.4–1.0 mEq/l and human brain levels of FLUO reach 10–15 µM during treatment for depression^60^. Note that FLUO is known to accumulate inside cells^61^, and we assume that the effects on inositol and PIns are due to intracellular FLUO (as are the effects of lithium intracellular). The cortical neurons we use do not produce serotonin, and so the FLUO effects we describe are independent of serotonin re-uptake. FLUO is marketed as the SSRI Prozac, but it has many effects in the brain in addition to its effects on serotonin re-uptake^62,63^ and it is possible that some of these are secondary to effects on PIns synthesis and PIP_2_ turnover.

In the treatment of BD, lithium is primarily an anti-manic drug although it also limits the frequency of mood swings, whereas FLUO acts as an antidepressant. The opposing effects of lithium and FLUO on the rate of PIns turnover – lithium inhibits and FLUO stimulates – has interesting implications for the nature of mood instability in BD. One possibility is that there is faulty regulation in bipolar individuals of PIP_2_ regulated vesicle recycling, which in turn regulates the activity of neuronal circuits involved in plasticity, including those involved in mood control. Our results with lithium and FLUO provide a neurochemical explanation of why these drugs can modulate mood swings in opposing ways. It is also possible that FLUO’s effects on PIns turnover contribute to its antidepressant action in unipolar depression but, more importantly, to its destabilizing effects on mood in bipolar individuals when prescribed without the balance of a mood stabilizer such as lithium or VPA^2,3^.

Taken together, our results suggest a new hypothesis for the consequences of lithium’s inhibition of IMPase in brain neurons. Because of the uncompetitive nature of this inhibition, IP_1_ accumulates in neuronal compartments where PIns turnover is high and we suggest that the accumulated IP_1_ competitively inhibits PI-synthase. The inositol reversibility of lithium’s effects could then be explained by an increase in inositol uptake leading to a reversal of IP_1_ inhibition of PI-synthase, rather than to alleviation of hypothetical ‘inositol depletion’. In addition, lithium indirectly inhibits uptake of inositol that is used for rapid PIns synthesis in the neurons. These two aspects of lithium’s effects on neurons could confine the inhibition of PIns synthesis to neurons that do not express the high capacity SMITs, and to compartments, such as synapses, where extracellular inositol may be limiting. We propose that such susceptible synapses include those in the neocortex involved in mood control.

In summary, our results show that PIns synthesis and turnover of PIP_2_ in neurons is a target for lithium and FLUO, which are the most frequently prescribed mood-stabilizing and antidepressant drugs, respectively. The finding that both drugs used at therapeutically relevant doses have effects that converge on the regulation of the rate of neuronal PIns synthesis suggests that PIns metabolism, as well as the uptake of inositol as sites for psychiatric drug action should be re-evaluated. Our results also suggest a possible explanation for the destabilizing effects of antidepressants in BD and point to dysregulation of PIns turnover at synapses within some neuronal circuits as a possible underlying cause of BD. In addition, our results raise unexpected questions about how neurons acquire inositol and open new avenues for investigation of how neurons regulate PIP_2_ turnover.

## Electronic supplementary material

Supplemental Text

Supplemental Figure 1

Supplemental Figure 2

Supplemental Figure 3

Supplemental Figure 4

Supplemental Figure 5
