# Supplementary material for: Lithium and fluoxetine regulate the rate of phosphoinositide synthesis in neurons: a new view of their mechanisms of action in bipolar disorder
Source: Transl Psychiatry. 2018 Aug 31;8:175. doi: 10.1038/s41398-018-0235-2 (PMC6119186; doi:10.1038/s41398-018-0235-2)
Supplement: Supplementary file 1 — Supplemental Text [file 41398_2018_235_MOESM1_ESM.docx]

**Supplemental material**

**Lithium and Fluoxetine regulate the rate of phosphoinositide synthesis in neurons: a new view of their mechanisms of action in bipolar disorder**

Adolfo Saiardi and Anne W Mudge

Medical Research Council Laboratory for Molecular Cell Biology and Neuroscience, Physiology, and Pharmacology, University College London, Gower Street, London, WC1E 6BT, UK

**SUPPLEMENTAL FIGURE LEGENDS**

**Figure S1. Electron micrographs of cortical neurons cultured for 11 days illustrating heterogeneity of intracellular compartments**

(a) Low power micrograph showing cell somas and a network of neural processes. (b) A neural process with a clathrin-coated pit. (c) A neural process with prominent microtubules, but few organelles. (d, e) Neural processes with abundant endoplasmic reticulum, mitochondria, and spine. (f, g, h) Areas of synaptic profiles with clusters of synaptic vesicles. Scale bars: (a) 10 µm, (b-g) 500 nm.

**Figure S2. SAX- HPLC profiles of inositol, IPs and PIns extracted from cortical neurons**

(a) Profiles of cytosolic inositol and IPs in acid extracts of cultured cortical neurons labelled to steady-state for 5 d (D6-11) with 10 µCi/ml ^3^H-inositol. The y-axes show the cpm / well of each chromatography fraction. The relative amounts of radioactivity in each component after steady-state labelling reflect the total relative amount of each component, because the neurons do not synthesize inositol.

(b) Profiles of lipid phosphoinositides extracted in organic solvents following acid extraction of the neurons used in (a). Lipid samples were processed for isolation of the glycerophosphoinositide head groups before chromatography.

**Figure S3. Changes in the relative amounts of IPs after stimulation of PIP_2_ hydrolysis**

(a) Changes in the relative amounts of IPs relative to controls (black line) following a 30 sec stimulation of neurons with 40 mM KCl (green line) or 1 mM carbachol (red line). Each line represents the mean of 2 samples. Note the large increases in IP_1_, IP_2_ and IP_3_ and that neither stimulus changed the levels of IP_6_.

(b) Changes in the relative amounts of IPs relative to controls (black line) following a 20 min stimulation of neurons with 40 mM KCl (green line) or 1 mM carbachol (red line). Each line represents the mean of 2 samples. Note that the large increases in IP_1_ (KCl and carbachol_)_ and IP_4_ (carbachol only) and that neither stimulus changed the levels of IP_6_.

**Figure S4. The rate of uptake of 3H-inositol and incorporation into IPs**

Neurons were exposed to ^3^H-inositol in the extracellular fluid for periods of 10 min, 1hr, and 3 hr as indicated. Panels on the left and right represent the same data displayed with 2 different scales. The data illustrate that uptake of ^3^H-inositol is linear over 3 hrs and that IP_1_ and IP_2_ from PIP_2_ hydrolysis are detectable at 10 min. The amounts of IP_1_ and IP_2_ increase over 1-3 hrs and IP_5_ is detected after 3 hrs. From this data, we decided to measure the rate of PIns synthesis by routinely measuring the amount of ^3^H-inositol incorporated into membrane PIns and the generation of IPs_(1, 2)_ after 1 hr as shown in Figure 3.

**Figure S5. Dose responses of lithium and FLUO on uptake of ^3^H-inositol and incorporation into lipid membranes**

Data show the radioactivity in the cytosol and lipid after a 1 hr exposure to ^3^H-inositol in the extracellular fluid. Neurons were treated with either lithium or FLUO for 20 hr prior to extractions as indicated. Data points are means +/- SEM, n=3.

**SUPPLEMENTAL METHODS**

Extractions

Cytosolic components were extracted from 6-well plates with 0.5 ml of 2 M perchloric acid for 5 min at 4° C, followed by a second extraction for 1 min; these extracts were combined. Membranes were then recovered from the same wells by scraping in 0.7 ml of collection solution (500μl MeOH/200μl H_2_O/20μl of 10 M HCl, containing fresh 1mM EDTA) and placing this in a 2 ml tube to which was added 500μl chloroform; after vortexing for 30 sec, we added 300 μl of water containing 3 mM of tetrabutylammoniumhydrogen sulphate (TBAHS; Sigma) and 10 μg/5μl of bovine brain polyphosphoinositides (Sigma). Tubes were vortexed for 30 sec, incubated on ice for 15 min and then centrifuged for 3 min at 5000 rpm. To further purify the lipid phosphoinositides, the chloroform layer was collected (carefully avoiding the insoluble interphase material) and washed with an equal volume of synthetic upper phase (10μl chloroform /480μl MtOH/470μl of HCl 0.6M containing 1mM EDTA). The tubes were vortexed for 30 second and then centrifuged for 3 minutes at 5000 rpm. The collected chloroform layers were dried under vacuum using an unheated Speedvac. Lipids were deacylated as previously described (Dove and Michell, 2009).

SAX-HPLC analysis

The 2M perchloric acid extracts were neutralized with potassium carbonate, which precipitated protein, and the acid-soluble inositol polyphosphates were resolved using SAX-HPLC with a Whatman PartiSphere SAX (4.6 × 125 mm) column as previously described (Azevedo and Saiardi, 2006). Isolated glycerophosphoinositides were resuspended in 1 mM EDTA, and resolved using SAX-HPLC with a Whatman PartiSphere SAX (4.6 × 250 mm) column. The column was eluted with a gradient generated by mixing 1 mM EDTA and buffer B [1 mM EDTA/1.3 M (NH_4_)_2_HPO_4_, pH 3.8] in one of two methods: (i) 0–5 min, 0% B; 5–105 min, 0–100% B; 105–120 min, 100% B; or (ii) 0–5 min, 0% B; 5–85 min, 0–50% B; 85–105 min, 50%–100% B; 105–120 min, 100% B. Spectrophotometric monitoring of AMP, ADP, and ATP elution times and authentic deacylated [^3^H]PI(4,5)P_2_ (PerkinElmer NEN) were used as standards. 1 ml fractions were collected and 4 ml of Ultima-Flo AP LCS-mixture (Packard) added before radioactivity per faction was analysed using a scintillation counter.
