# Supplementary figures and images for: Lithium and fluoxetine regulate the rate of phosphoinositide synthesis in neurons: a new view of their mechanisms of action in bipolar disorder
Source: Transl Psychiatry. 2018 Aug 31;8:175. doi: 10.1038/s41398-018-0235-2 (PMC6119186; doi:10.1038/s41398-018-0235-2)

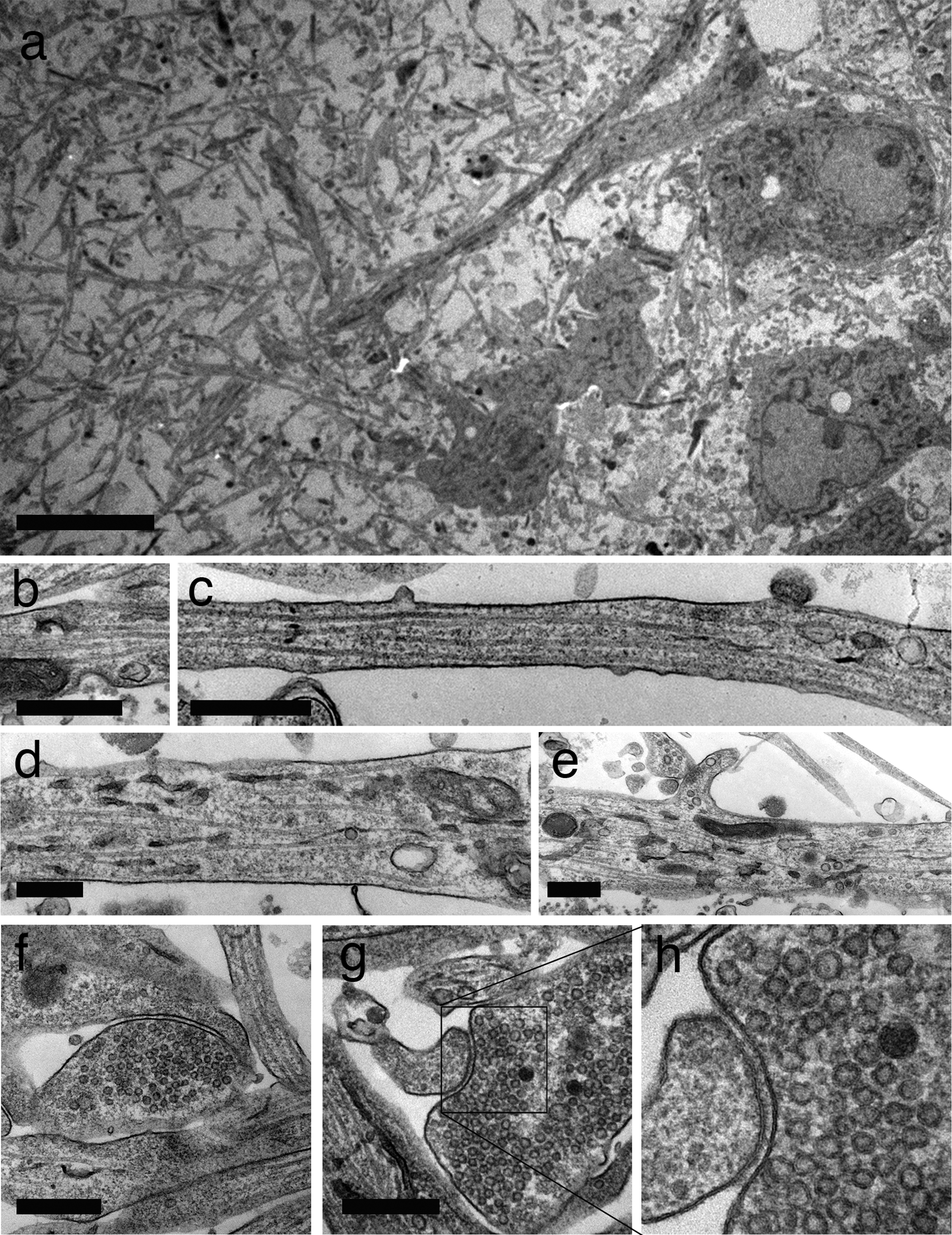

Supplement: Supplementary file 2 — Supplemental Figure 1 [file 41398_2018_235_MOESM2_ESM.tif]

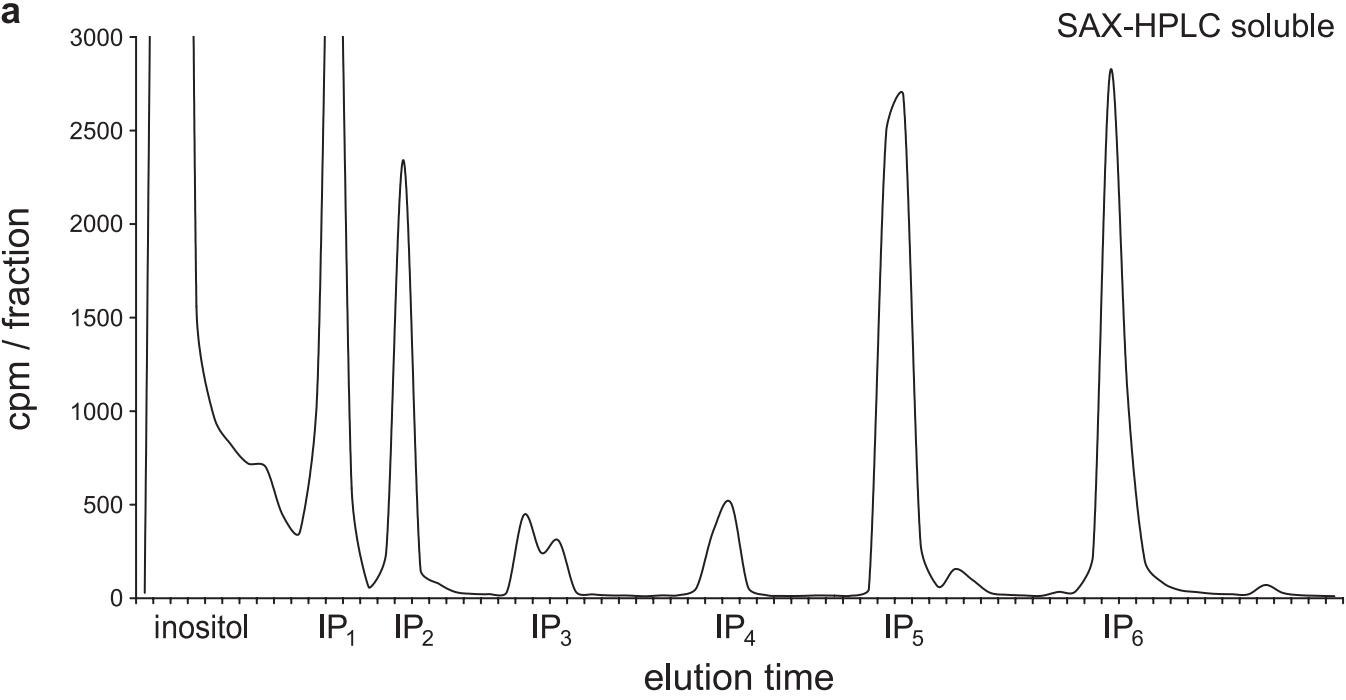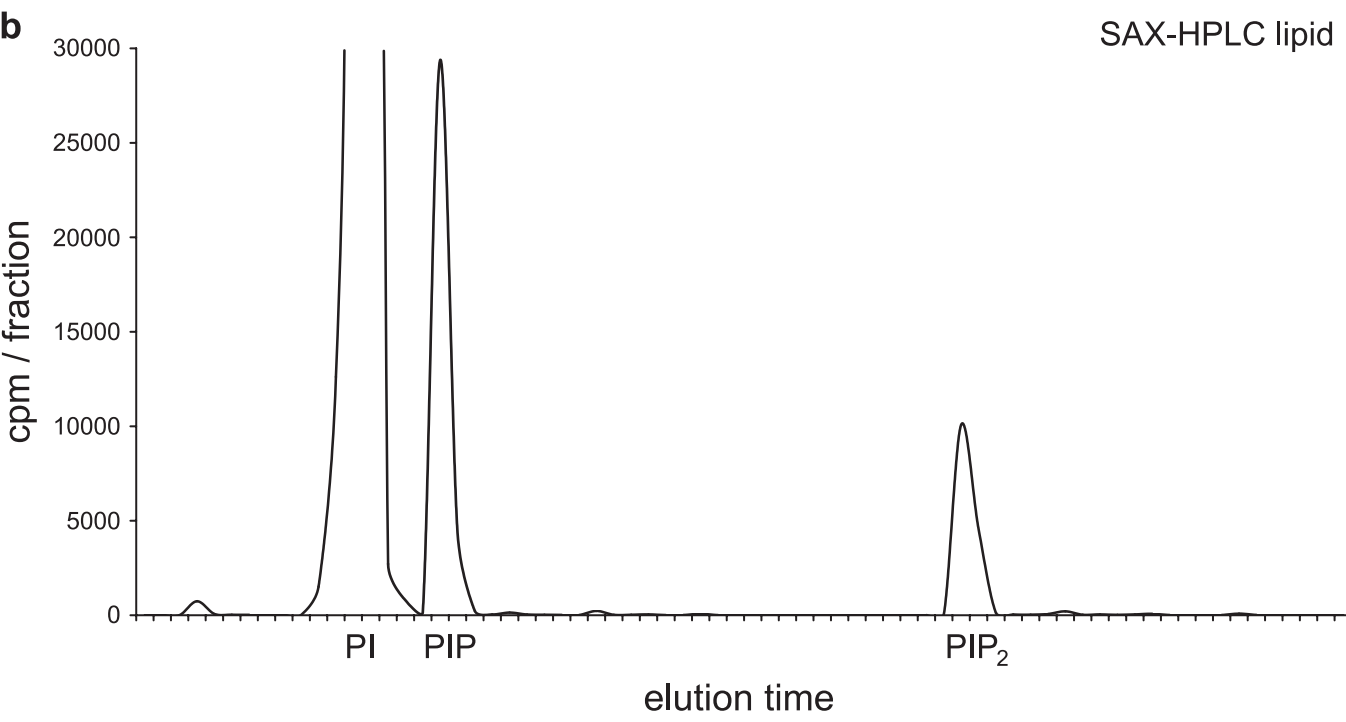

Supplement: Supplementary file 3 — Supplemental Figure 2 [file 41398_2018_235_MOESM3_ESM.pdf]

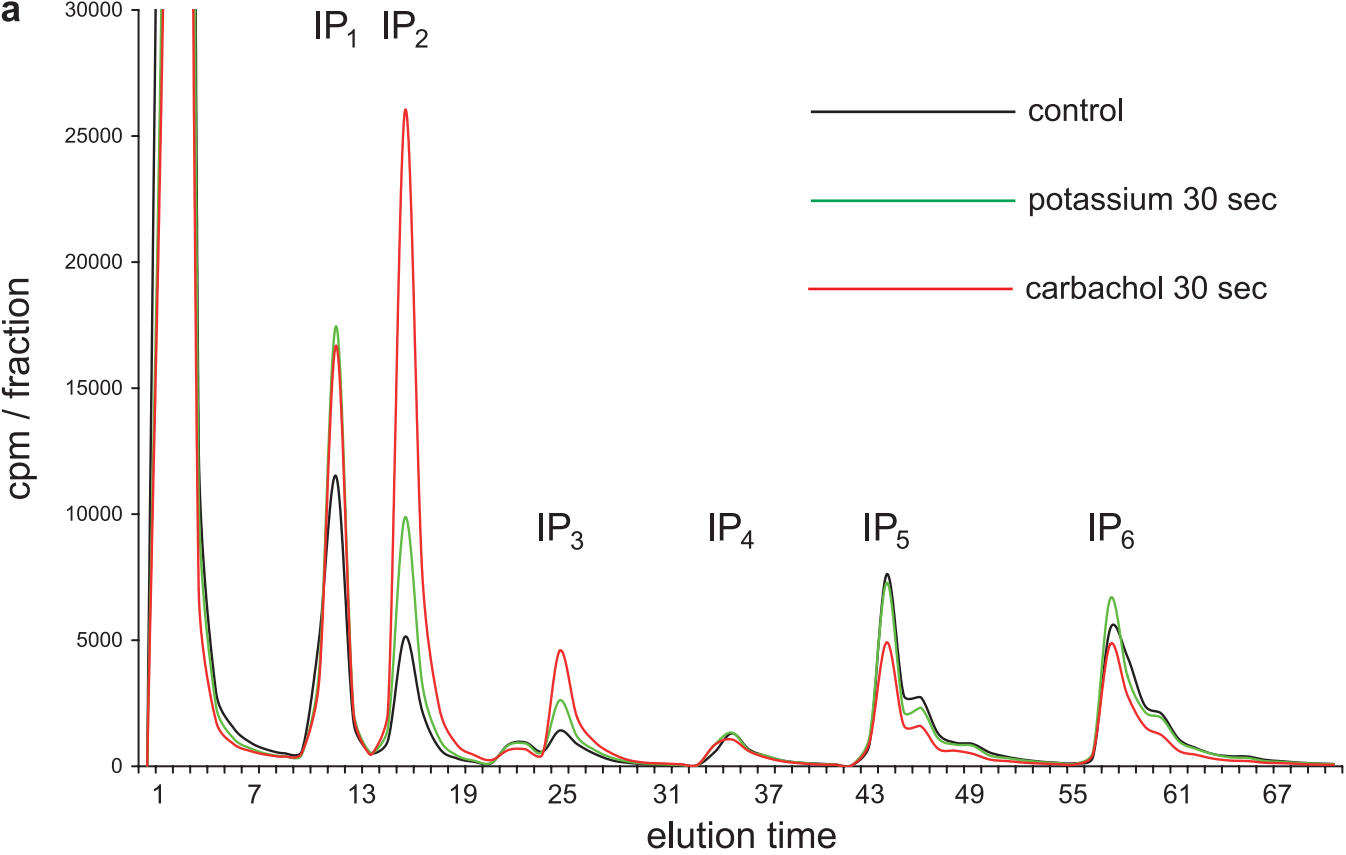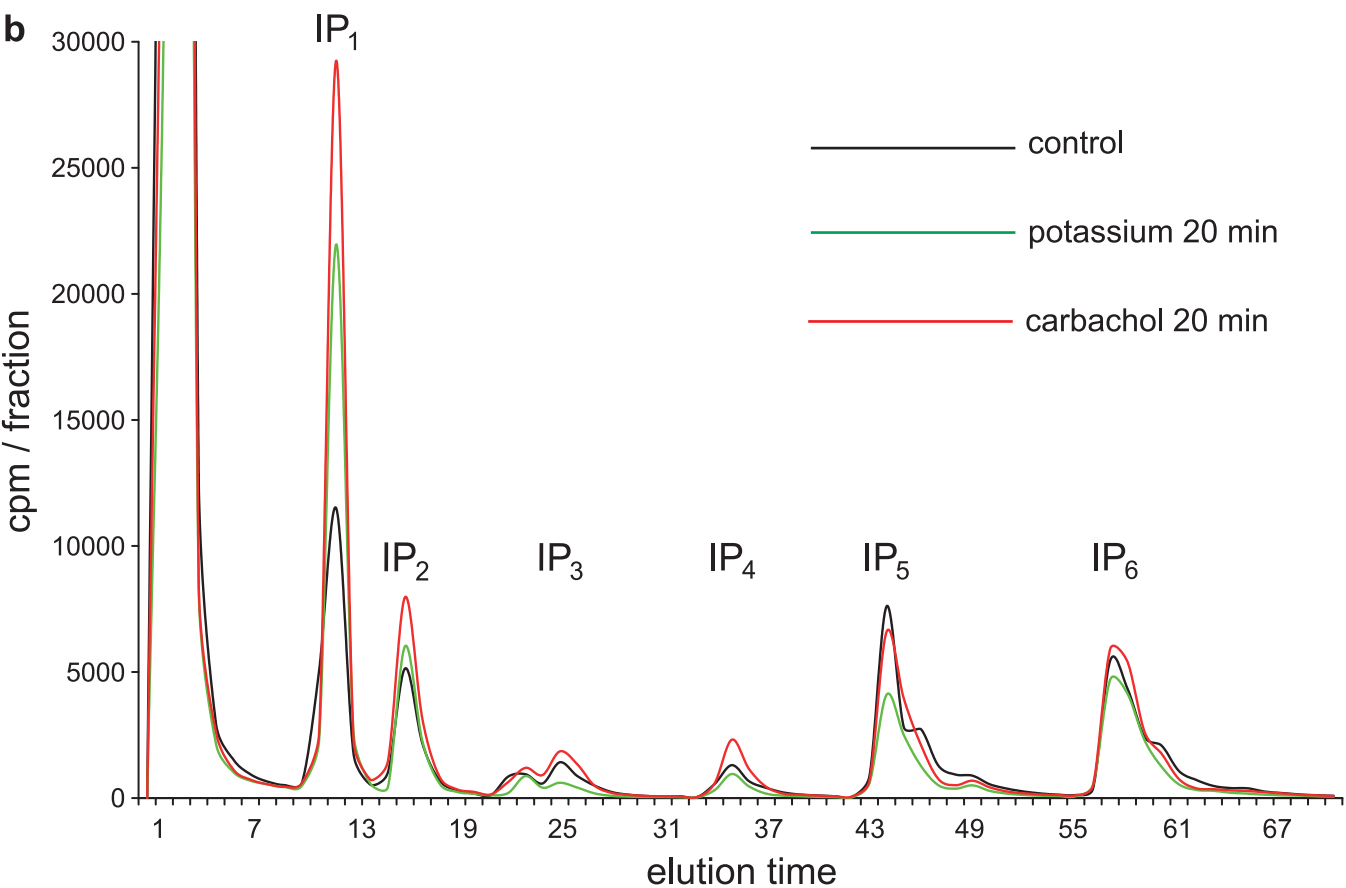

Supplement: Supplementary file 4 — Supplemental Figure 3 [file 41398_2018_235_MOESM4_ESM.pdf]

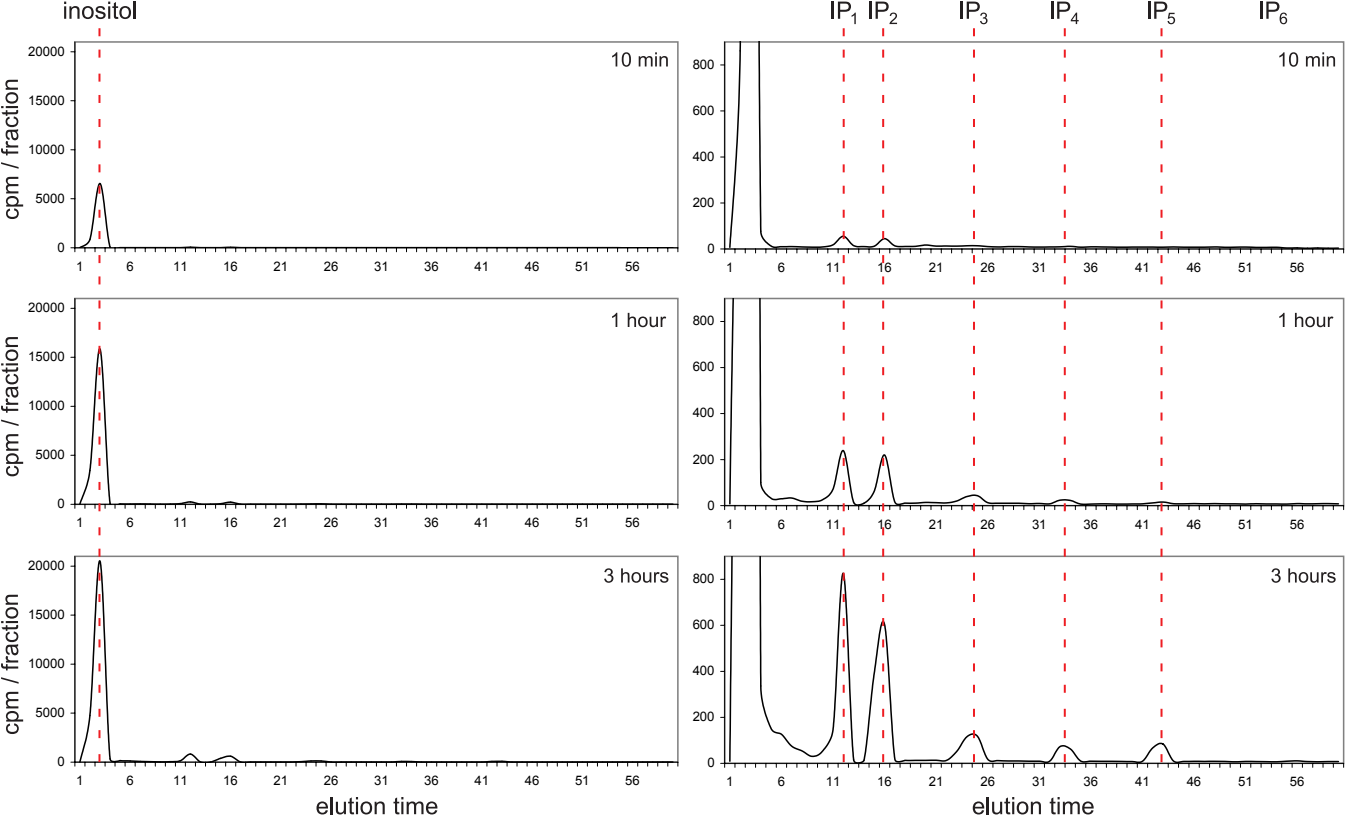

Supplement: Supplementary file 5 — Supplemental Figure 4 [file 41398_2018_235_MOESM5_ESM.pdf]

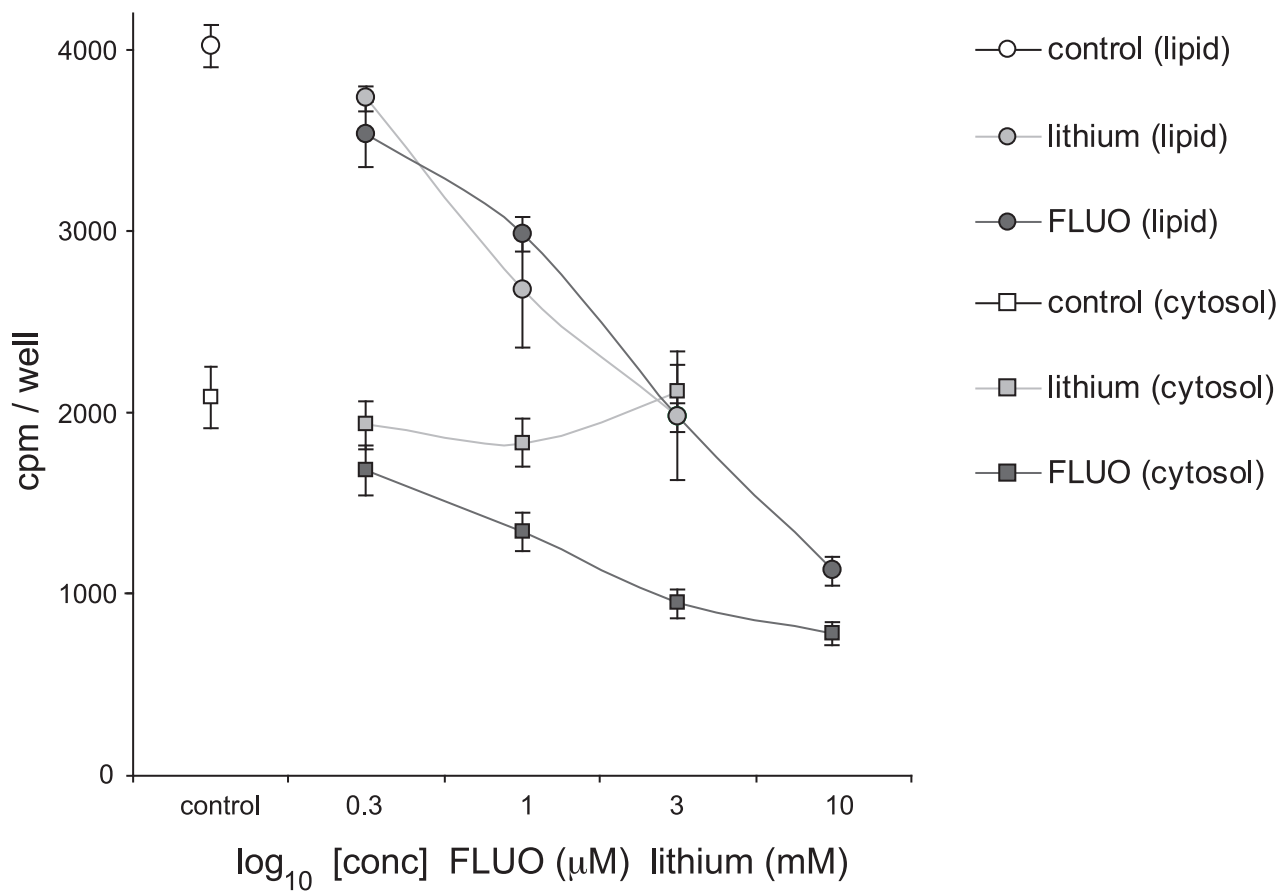

Supplement: Supplementary file 6 — Supplemental Figure 5 [file 41398_2018_235_MOESM6_ESM.pdf]
